# Aspirin for primary prevention in the elderly

**DOI:** 10.18632/aging.102255

**Published:** 2019-09-06

**Authors:** Marwan Saad, Hesham K. Abdelaziz, Jawahar L. Mehta

**Affiliations:** 1 Cardiovascular Institute, Warren Alpert Medical School of Brown University, Providence, RI 02903, USA; 2 Lancashire Cardiac Center, Blackpool Victoria Hospital, Blackpool, UK; 3 Division of Cardiovascular Medicine, University of Arkansas for Medical Sciences, The Central Arkansas Veterans Healthcare System, Little Rock, AR 72205, USA

**Keywords:** primary prevention, aspirin, myocardial infarction, bleeding, mortality

Aspirin remains among the most widely used medications across the globe, not only for secondary prevention but also for primary prevention of cardiovascular disease (CVD), with nearly 36 million adults without prior CVD event taking aspirin in the US alone [1]. Since its approval for secondary prevention of CVD by the FDA in 1985, multiple randomized controlled trials have examined the role of aspirin in primary prevention. While early trials showed benefit of aspirin in reducing CV events, more recent trials have challenged these findings with even a signal towards net harm. This has resulted in change in the most recent U.S. Preventive Services Task Force recommendations towards a shared decision making especially with patients 60-70 years who are at CVD risk [2].

In 2018, 3 pivotal trials raised questions regarding benefits of aspirin in primary prevention, leading to a new update in the preventive care guidelines from the AHA/ACC [3]. The latest meta-analysis of 15 RCTs with a total of 165,502 participants conducted by us confirmed lack of mortality benefit of aspirin in primary prevention, and the observed benefit of aspirin in reducing non-fatal CVD events was negated by a higher rate of non-fatal bleeding events. Furthermore, incidence of cancer or cancer mortality did not seem to be reduced with aspirin over 6.5 years of follow-up [4].

The major advancements in healthcare have led to a dramatic increase in life expectancy over the past century with a substantial demographic shift toward aging of population. It has been shown that the risk of CVD doubles with each decade of life independent of traditional risk factors [5]. This has resulted in CVD being the principal cause of disability and death in the elderly; hence, primary interventions for such CVD have become a high priority.

Aspirin use for secondary prevention in all populations has been widely accepted, as the benefits linked to reduction in myocardial infarction and stroke are likely to overweigh the risk of major bleeding. However, as shown in our meta-analysis the risk-benefit has not been favorable in primary prevention trials. Of note, while no major trial exclusively enrolled elderly population, except for the Effect of Aspirin on Cardiovascular Events and Bleeding in the Healthy Elderly (ASPREE) trial [6], subgroup analyses from major trials demonstrated minimal heterogeneity in CV outcomes based on age (< 65 vs > 65 years) [4,7,8].

In ASPREE trial conducted exclusively in healthy adults ≥70 years of age (or ≥65 years of age among blacks and Hispanics), low-dose aspirin not only failed to lower the risk of CVD, it significantly increased the risk of major bleeding events. Aspirin also failed to show any benefit in terms of disability-free survival [7].

There is a greater bleeding risk with increasing age, with about 50% increase in the risk of hemorrhagic stroke and nearly double the risk of major extracranial bleeding with each decade of age regardless of aspirin use. Therefore, it is important to quantify the relative risk reduction of CVD events and balance it with higher bleeding risk. Elderly patients also tend to have multiple comorbidities, as well as chronic pain leading to relatively higher consumption of over-the-counter non-steroidal anti-inflammatory drugs (NSAIDs) that can increase the risk of gastrointestinal (GI) bleeding either through mucosal irritation or potentiation of aspirin’s antiplatelet effect.

Another area of debate with aspirin is the proposed benefit in reducing cancer especially colorectal cancer. While prior studies suggested possible reduction in cancer incidence and cancer-related deaths, recently studies failed to confirm this phenomenon [4,7,8]. Importantly, contemporary studies examined the effect of aspirin over a relatively short follow-up period that may not be adequate to assess impact on cancer risk. Previous reports suggested that the potential beneﬁt of aspirin in colorectal cancer reduction may not be apparent until 10 years after initiating aspirin therapy. This is an important point to consider in elderly who may not have enough life expectancy to experience such potential benefit of aspirin.

The current guidelines recommend against the use of aspirin for primary prevention in adults >70 years of age as the risk of major bleeding is likely to overweigh the potential benefit in reducing CV events, and the less likelihood to observe the expected benefit of reducing cancer risk [3]. With the current data, it may be reasonable to focus on other primary prevention strategies for CVD and to tailor the use of aspirin, especially in the elderly, to individual persons based on their CVD versus bleeding risk. In the elderly, multiple factors can determine bleeding risk including prior history of GI bleeding, liver or renal disease, fall risk, frailty, and concomitant use of anticoagulants including NSAIDs. Further, based on results of meta-analysis it is prudent that only low-dose aspirin should be utilized if aspirin is deemed necessary [4]. The use of enteric coated aspirin and concurrent proton pump inhibitors use may also be considered to reduce the risk of GI bleeding.

Various other questions regarding aspirin use still exist. Should individuals who are taking aspirin and have reached 70 years of age without any adverse effects continue aspirin therapy? Obviously, they should discuss with their clinician, and decision be based on their overall CVD risk and personal preferences. Also, should aspirin use be continued for secondary prevention in patients who have done well for years after their CV event as “life-long therapy”, especially in subjects ≥70 years of age? We certainly do not have answers for this question. Perhaps future studies will address this important question.

In summary, the use of aspirin for primary prevention of CVD and cancer is debatable, especially in the elderly population. Health education is recommended to reduce the risk of complications associated with “over-the-counter” use of aspirin for primary prevention.
